# Immunogenicity of Individual Vaccine Components in a Bivalent Nicotine Vaccine Differ According to Vaccine Formulation and Administration Conditions

**DOI:** 10.1371/journal.pone.0082557

**Published:** 2013-12-02

**Authors:** Katherine E. Cornish, Sabina H. L. de Villiers, Marco Pravetoni, Paul R. Pentel

**Affiliations:** 1 Department of Pharmacology, University of Minnesota, Minneapolis, Minnesota, United States of America; 2 Minneapolis Medical Research Foundation, Minneapolis, Minnesota, United States of America; 3 Center for Global Studies and Social Responsibility, University of Minnesota, Minneapolis, Minnesota, United States of America; 4 Department of Physiology and Pharmacology, Karolinska Institutet, Stockholm, Sweden; 5 Department of Medicine, University of Minnesota, Minneapolis, Minnesota, United States of America; The Scripps Research Institute, United States of America

## Abstract

Structurally distinct nicotine immunogens can elicit independent antibody responses against nicotine when administered concurrently. Co-administering different nicotine immunogens together as a multivalent vaccine could be a useful way to generate higher antibody levels than with monovalent vaccines alone. The immunogenicity and additivity of monovalent and bivalent nicotine vaccines was studied across a range of immunogen doses, adjuvants, and routes to assess the generality of this approach. Rats were vaccinated with total immunogen doses of 12.5 - 100 μg of 3′-aminomethyl nicotine conjugated to recombinant Pseudomonas exoprotein A (3′-AmNic-rEPA), 6-carboxymethylureido nicotine conjugated to keyhole limpet hemocyanin (6-CMUNic-KLH), or both. Vaccines were administered s.c. in alum or i.p. in Freund’s adjuvant at matched total immunogen doses. When administered s.c. in alum, the contributions of the individual immunogens to total nicotine-specific antibody (NicAb) titers and concentrations were preserved across a range of doses. Antibody affinity for nicotine varied greatly among individuals but was similar for monovalent and bivalent vaccines. However when administered i.p. in Freund’s adjuvant the contributions of the individual immunogens to total NicAb titers and concentrations were compromised at some doses. These results support the possibility of co-administering structurally distinct nicotine immunogens to achieve a more robust immune response than can be obtained with monovalent immunogens alone. Choice of adjuvant was important for the preservation of immunogen component activity.

## Introduction

Vaccination is being studied as a treatment for drug addiction. Immunization with drug hapten-protein conjugate vaccines produces drug-specific antibodies that bind and sequester drug in serum, preventing or slowing drug distribution to brain and attenuating drug-induced behavioral effects [1–3]. 

Vaccines against nicotine and cocaine have entered clinical trials and immunotherapies against opioids, methamphetamine, and phencyclidine have shown efficacy in animal models [4–9]. The efficacy of these vaccines in both animals and humans is closely correlated with the level of serum drug-specific antibodies produced. In several clinical trials of nicotine vaccines, the top third of subjects with the highest serum nicotine-specific antibody (NicAb) concentrations or titers had higher smoking cessation rates than controls [8,10]. However there was no difference in smoking cessation rates when all subjects were included in the analyses because of the overall low and variable NicAb levels [8,10,11]. Reliable production of sufficient NicAb levels appears to be the primary challenge for successfully translating vaccination against nicotine into clinical use. 

A potential strategy for producing a more robust immune response is to co-administer two or more nicotine immunogens. Combining immunogens in a multivalent vaccine is a well-established approach for preventing infectious diseases [12,13]. Multiple immunogens are regularly combined into multivalent vaccines with little to no compromise in the immunogenicity of each individual component. The goal when combining vaccines for infectious diseases is to achieve a broad specificity, such as the targeting of several serotypes in the influenza vaccine. Multivalent vaccines also allow administration of several unrelated immunogens at once for convenience, such as diphtheria, tetanus, and pertussis. In contrast, the goal in extending this multivalent approach to nicotine is to achieve a greater response to the single target of nicotine. In a previous study, the nicotine immunogens 3′-aminomethyl nicotine conjugated to recombinant Pseudomonas exoprotein A (3′-AmNic-rEPA) and 6-carboxymethylureido nicotine conjugated to keyhole limpet hemocyanin (6-CMUNic-KLH) were studied in rats to evaluate the potential cross-reactivity of their elicited antibodies [14]. These immunogens differed in the site of linker attachment to nicotine, linker composition, and carrier protein. Both immunogens elicited high concentrations of antibodies against nicotine in rats but with different hapten specificities. The 3′-AmNic-rEPA immunogen produced antibodies that recognized the 3′-AmNic hapten with <10% cross-reactivity for 6-CMUNic conjugates, and vice versa; this indicates that these two immunogens function as independent epitopes, activating largely non-overlapping populations of B cells. Antibodies against both 3′-AmNic and 6-CMUNic haptens were generated when co-administered as a bivalent vaccine. These preliminary data suggested that 3′-AmNic-rEPA and 6-CMUnic-KLH might be suitable for combined use as a means of enhancing the immune response to nicotine. Because these initial studies were carried out using Freund’s adjuvant, which is not suitable for human use, and at only one vaccine dose size, further evaluation was undertaken in the current study to explore the clinical potential of concurrent administration of these immunogens.

The current study examines this approach in order to assess its generality. Monovalent and bivalent nicotine vaccines formulated from the 3′-AmNic-rEPA and 6-CMUnic-KLH immunogens were compared in rats across a range of matched total immunogen doses administered either s.c. in alum or i.p. in Freund’s adjuvant. Administration s.c. in alum was studied because of its clinical relevance, and administration i.p. in Freund’s was studied because of its common use in animal models. The primary goals of this study were to determine whether the bivalent vaccine generated higher NicAb titers or concentrations compared to monovalent vaccines and also whether the individual vaccine components retained their immunogenicity when combined under these conditions. A secondary goal was to examine individual variability in nicotine binding affinity since this measure of antibody quality has not been well characterized for nicotine vaccines.

## Materials and Methods

### 2.1: Animals

#### 2.1.1: Ethics Statement

This study was carried out in strict accordance with the recommendations in the Guide for the Care and Use of Laboratory Animals of the National Institutes of Health. The protocol was approved by the Minneapolis Medical Research Foundation Institutional Animal Care and Use Committee (Protocol Number: 07-11). All animal sacrifice was performed under Innovar (fentanyl/droperidol) anesthesia, and all efforts were made to minimize suffering.

#### 2.1.2: Animal Care

Male Holtzman Sprague Dawley rats (Harlan, Indianapolis, IN) weighing 275-300 g at time of arrival were double-housed in temperature- and humidity-controlled rooms and maintained on a 12 h light/dark cycle. Animals received unrestricted water and food. 

### 2.2: Study Design and Vaccination Protocol

Two separate, parallel experiments were performed; the first experiment administered vaccines s.c. in alum and the second experiment administered vaccines i.p. in Freund’s adjuvant. In the first experiment, all vaccines contained 0.2 ml of immunogen(s) in PBS and 0.2 ml of alum (Alhydrogel [Al(OH)_3_], E. M. Sergeant Chemical Co., Clifton NJ ), yielding a final injection volume of 0.4 ml and a final aluminum concentration of 1.25 mg/ml. When two immunogens were included in a dose, they were added to alum at the same time. Tubes containing vaccines were gently inverted and allowed to sit at room temperature for 30 minutes. Rats were vaccinated every three weeks (days 0, 21, 42, 63) for a total of 4 immunizations. Vaccines were administered s.c. in the upper back. On day 70, rats were anesthetized with Innovar (fentanyl/droperidol), sacrificed, and trunk blood was obtained for ELISA and equilibrium dialysis.

Groups of 12 rats received 3′-AmNic-rEPA alone, 6-CMUNic-KLH alone, or both (in the bivalent vaccine), each at total immunogen doses of 12.5 µg, 25 µg, 50 µg, or 100 µg for a total of 12 groups (see Table 1). In the bivalent groups, the total immunogen dose refers to the sum of individual immunogen doses used (i.e. 6.25 µg of 3′-AmNic-rEPA + 6.25 µg of 6-CMUNic-KLH = 12.5 µg total immunogen dose). 

**Table 1:** Group design.

**Vaccine**	**3'-AmNic-rEPA**	**6-CMUNic-KLH**
	12.5μg	--
Monovalent	25 μg	--
3'-AmNic-rEPA	50 μg	--
	100 μg	--
	--	12.5 μg
Monovalent	--	25 μg
6-CMUNic-KLH	--	50 μg
	--	100 μg
	6.25 μg	6.25 μg
Bivalent	12.5 μg	12.5 μg
	25 μg	25 μg
	50 μg	50 μg

In the second experiment, equal volumes of immunogen(s) in PBS and Freund’s adjuvant were combined for a final injection volume of 0.4 ml. Freund’s complete adjuvant (EMD Millipore, Billerica MA) was used for the first immunization and Freund’s incomplete adjuvant (Sigma-Aldrich, St. Louis MO) was used for subsequent immunizations. As with vaccines administered s.c. in alum, when two immunogens were included for bivalent vaccine preparation, they were added to Freund’s adjuvant at the same time. Tubes containing vaccines were vortexed for 10 minutes prior to injection. Vaccines were administered i.p. The vaccination protocol and experimental design was otherwise identical to that of the first experiment (Table 1), which utilized alum as an adjuvant.

### 2.3: Immunogens

**Figure 1:**
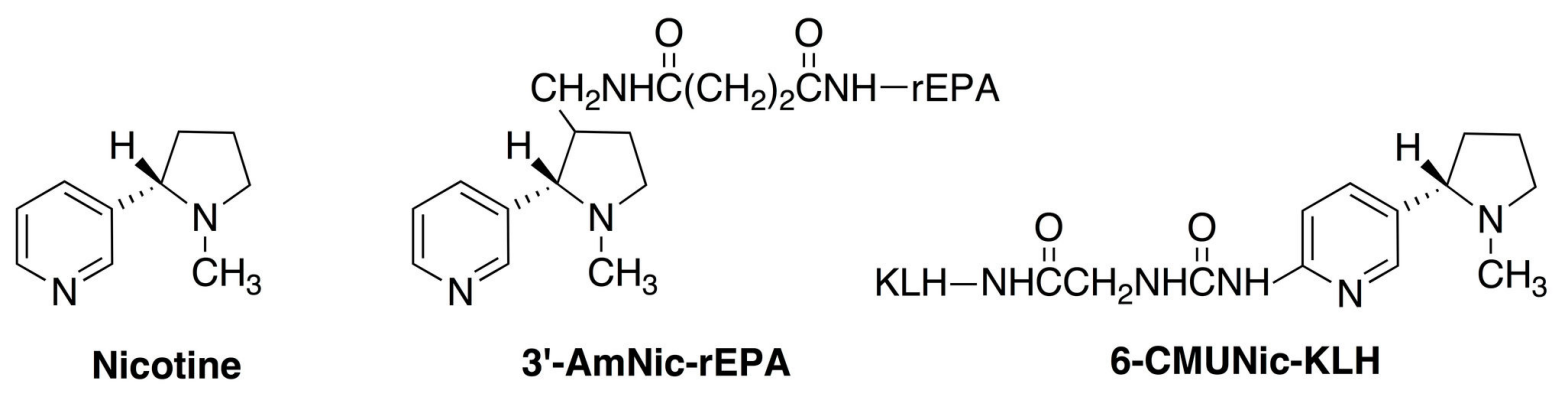
Nicotine Haptens 3′-AmNic-rEPA and 6-CMUNic-KLH.

The nicotine immunogen 3′-AmNic-rEPA consists of 3′-aminomethyl-nicotine conjugated to the carrier protein recombinant *Pseudomonas* exoprotein A (see Figure 1). Antibodies produced by 3′-AmNic-rEPA have a high affinity for nicotine (K_d_= 10-19 nM) and marginal (<1%) cross-reactivity with similar compounds including acetylcholine, nicotine metabolites cotinine and nicotine-*N*-oxide, and other neurotransmitters [15,16]. The nicotine immunogen 6-CMUNic-KLH consists of 6-carboxymethylureido nicotine conjugated to the carrier protein keyhole limpet hemocyanin (see Figure 1) [17]. Antibodies that are generated by 6-CMUNic-KLH also have a high affinity for nicotine (K_d_= 29-71 nM) and a specificity for nicotine similar to that of 3′-AmNic-rEPA [18,19]. 

### 2.4: Enzyme-linked Immunosorbent Assay (ELISA)

Serum nicotine-specific antibody (NicAb) concentrations and titers produced by vaccination were measured by ELISA using 3′-AmNic-polyglutamate or 6-CMUNic-BSA as the coating antigens and goat anti-rat horseradish peroxidase as the detecting antibody [20]. NicAb concentrations were measured against a standard curve derived from previously characterized serum NicAb samples. Cross-reactivity in the ELISA results was determined by measuring 3′-AmNic-rEPA-generated antibodies against the 6-CMUNic-BSA coating antigen and measuring 6-CMUNic-KLH-generated antibodies against the 3′-AmNic-polyglutamate coating antigen [14]. Cross-reactivity between the two immunogens across all vaccination conditions was less than 10%. The cross-reactivity from these assays was used to correct serum NicAb concentrations in the bivalent group. 

### 2.5: Equilibrium Dialysis

Equilibrium dialysis was used to measure antibody affinity. Six of 12 serum samples from each vaccine group receiving a total immunogen dose of 12.5 or 100 µg were analyzed. Nic311, a previously characterized IgG1_κ_ monoclonal antibody derived from immunization of mice with 3′-AmNic-rEPA served as a positive control [21,22]. Serum samples from vaccinated rats were diluted to a NicAb concentration of approximately 100 µg/ml and added to the serum side of 96-well equilibrium dialysis plates (MWCO 10 kDa; the Nest Group Inc, Southborough MA). The dialysate side consisted of non-immune (blank) rat serum containing unlabeled (-)-nicotine hydrogen tartrate salt (Sigma-Aldrich, St. Louis MO) at concentrations of 1-1024 nM with ^3^H-nicotine (81.7 µCi/ml; PerkinElmer, Boston MA). Plates were placed on a shaker for 72 hours at room temperature. The radiolabeled nicotine was used to estimate nicotine concentrations in serum and dialysate. Nicotine-binding affinity of NicAbs (represented by antibody dissociation constant K_d_) was measured from saturation curves using Prism 6.0 (GraphPad Software Inc., La Jolla CA). 

### 2.6: Statistical Analyses

Effects of vaccine group and total immunogen dose were examined using two-way ANOVA with vaccine group and dose as factors, followed by Holm-Sidak’s post-test. One-way ANOVA followed by Bonferroni’s post-test was used to explore within-group comparisons. The relationship between serum NicAb concentrations generated by individual immunogens within the bivalent group was examined using correlation analysis.

## Results

### 3.1: Experiment 1: Vaccines administered s.c. in alum adjuvant

#### 3.1.1: Serum antibody titers (Figure 2A)

**Figure 2:**
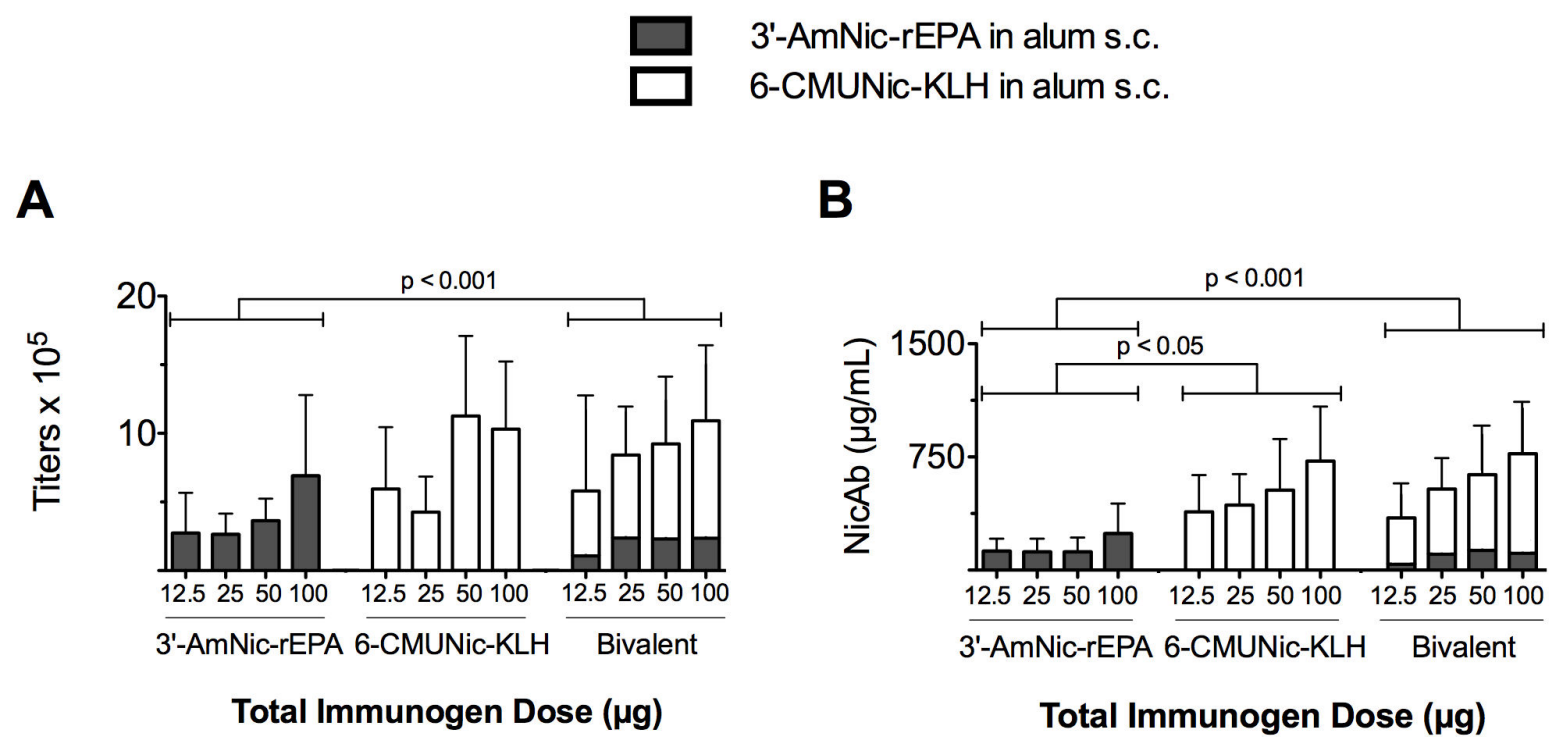
Serum nicotine-specific antibody (NicAb) titers and concentrations of rats immunized with monovalent or bivalent vaccines s.c. in alum (mean ± SD). Immunogen doses are the total administered, i.e. 100 µg bivalent = 50 µg 3′-AmNic-rEPA and 50 µg 6-CMUNic-KLH. In the bivalent group, the NicAb titers (A) or concentrations (B) generated by 3′-AmNic-rEPA (dark bars) and those generated by 6-CMUNic-KLH (white bars) are stacked to represent the total combined NicAb titer or concentration. The bivalent group achieved significantly higher total NicAb titers and concentrations than the group receiving 3′-AmNic-rEPA alone, but not compared to 6-CMUNic-KLH alone.

The analysis corresponding to Figure 1 considered the total dose of immunogen administered to each group. For example, rats in the 25 µg bivalent group received 12.5 µg of each individual immunogen. Total serum nicotine-specific antibody (NicAb) titer or concentration for the bivalent group refers to the sum of antibody titers or concentrations directed against 3′-AmNic-rEPA and 6-CMUNic-KLH. 

There was a main effect of vaccine group and of immunogen dose on total NicAb titers (p < 0.001 for both). When compared within each vaccine group, total NicAb titers increased with increasing total immunogen dose. Total NicAb titers in the bivalent group were significantly higher than those in the monovalent 3′-AmNic-rEPA group (p < 0.001) but did not differ from NicAb titers in the monovalent 6-CMUNic-KLH group. 

#### 3.1.2: Serum antibody concentrations (Figure 2B)

NicAb concentrations generally paralleled the NicAb titers. There was a main effect of both vaccine group and immunogen dose on total NicAb concentrations (p < 0.001 for both). Similar to the NicAb titers, when compared within each vaccine group, NicAb concentrations increased with increasing total immunogen dose. Total NicAb concentrations in the bivalent group were significantly higher than those in the monovalent 3′-AmNic-rEPA group (p < 0.001) but did not differ from those in the monovalent 6-CMUNic-KLH group. Serum NicAb concentrations in the 6-CMUNic-KLH group were higher than those of the monovalent 3′-AmNic-rEPA group (p < 0.05). 

In this analysis of total immunogen dose, the bivalent group received only half of the dose of each individual immunogen compared to its monovalent counterpart (i.e. 25 µg of bivalent = 12.5 µg of 3′-AmNic-rEPA and 12.5 µg of 6-CMUNic-KLH). Additional analyses (below) were therefore performed to provide a measure of whether the immunogenicity of the individual immunogens was retained when they were co-administered as components of the bivalent vaccine.

#### 3.1.3: Contributions of individual components in bivalent group (Figure 3)

**Figure 3:**
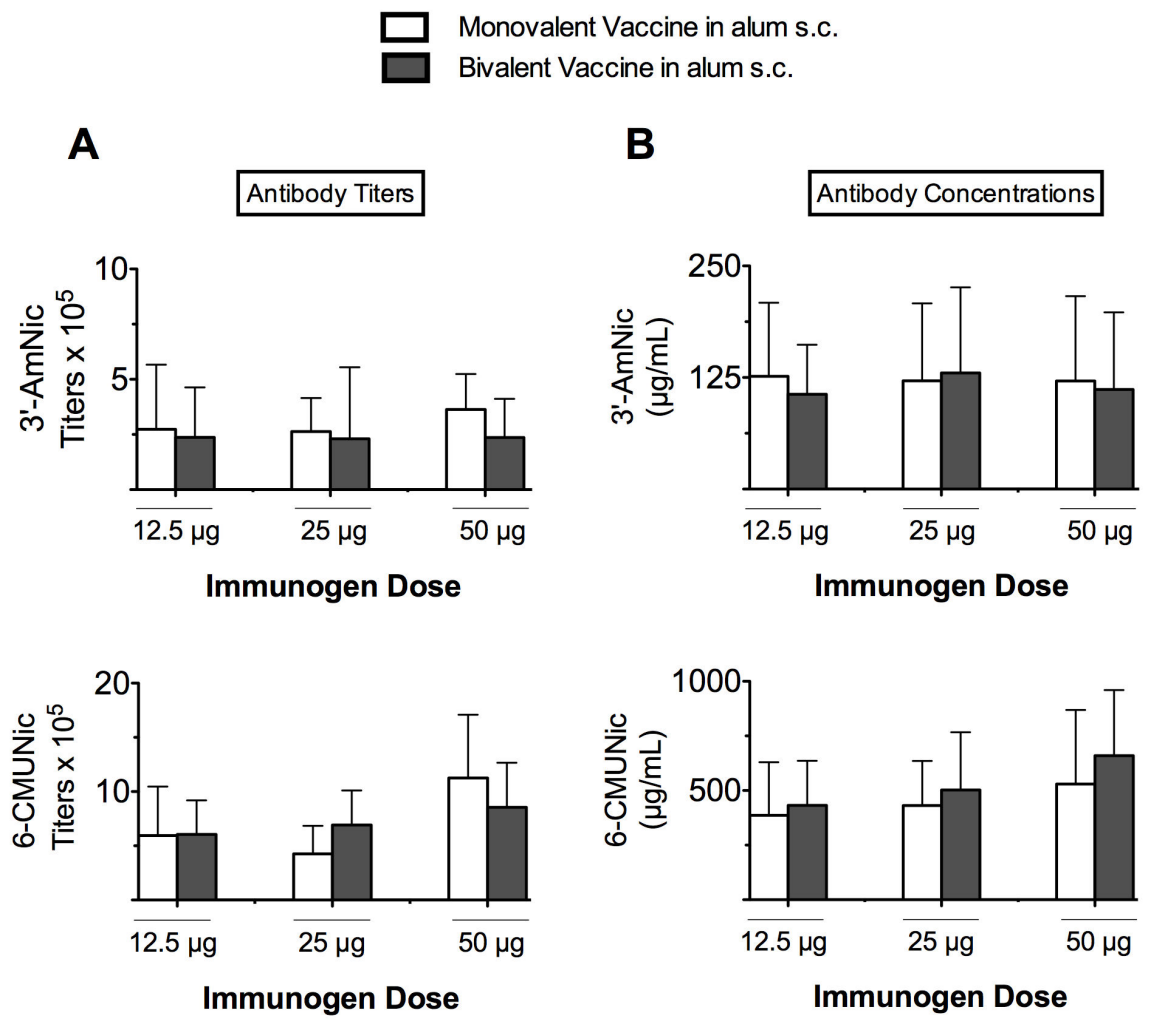
Nicotine-specific antibody (NicAb) titers and concentrations in monovalent vaccine groups and their individual dose-matched contributions when administered as a component of the bivalent vaccine (mean ± SD). Vaccines were administered s.c. in alum. Vertical axis indicates the hapten toward which the antibodies are directed. Note that the scales of vertical axes differ. The immunogenicity of both 3′-AmNic-rEPA and 6-CMUNic-KLH immunogens were retained when combined; there were no differences in hapten-specific NicAb titers (A) or concentrations (B) between the monovalent vaccine and bivalent vaccine groups.

This analysis compared matched individual immunogen doses when each immunogen was administered alone or as a component of the bivalent vaccine in rats immunized s.c. in alum. For example, NicAb titers generated by 12.5 µg of monovalent 3′-AmNic-rEPA vaccine were compared to the NicAb titers generated by 12.5 µg of 3′-AmNic-rEPA when delivered as a component of the 25 µg bivalent vaccine. In this analysis NicAb titers and concentrations, for both immunogens, did not differ regardless of whether immunogens were given alone or combined in the bivalent vaccine. That is, the immunogenicity of the individual immunogen components of the bivalent vaccine was preserved when they were co-administered in alum and administered s.c.

#### 3.1.4: Correlations among antibody titers or concentrations within the bivalent vaccine group (Figure 4)

**Figure 4:**
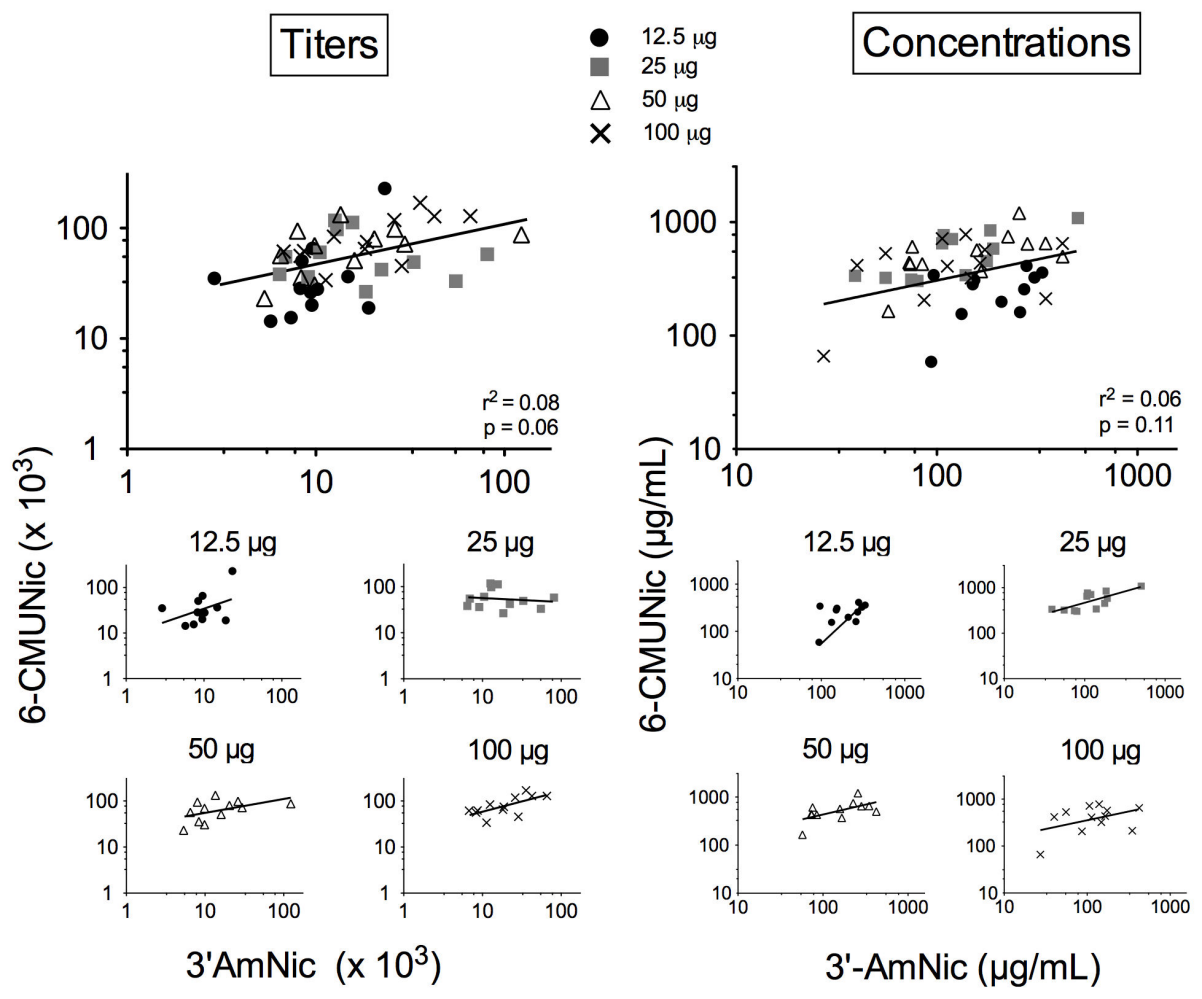
Correlations between serum NicAb levels produced by 3′-AmNic-rEPA and by 6-CMUNic-KLH in rats immunized with bivalent vaccine s.c. in alum. For all dose groups together (top panels), there were no significant correlations between the titers (A) or concentrations (B) attributable to 3′-AmNic-rEPA and those attributable to 6-CMUNic-KLH. When individual immunogen doses were analyzed separately, correlations between antibodies generated by 3′-AmNic-rEPA and 6-CMUNic-KLH were significant for titers at a dose of 100 µg (p = 0.02) and for concentrations at doses of 25 µg (p = 0.01) and 50 µg (p = 0.04). One data point was excluded from the top panel of concentrations to aid in representation of data because the 6-CMUNic-KLH concentration was 0 µg/ml but this point was included in the statistical analysis.

Analyzing all dose sizes together (Figure 4, top panels) there were no significant correlations between the serum NicAb titers (r^2^ = 0.08, p = 0.06) or concentrations (r^2^ = 0.06, p = 0.11) produced by 3′-AmNic-rEPA and 6-CMUNic-KLH when co-administered in the bivalent vaccine, although the p value of 0.06 was marginal for the titer correlation. When the different immunogen doses were analyzed separately, some doses showed a significant relationship between antibody titers or concentrations produced by the two immunogens.

#### 3.1.5: Antibody affinity (Figure 5A)

**Figure 5:**
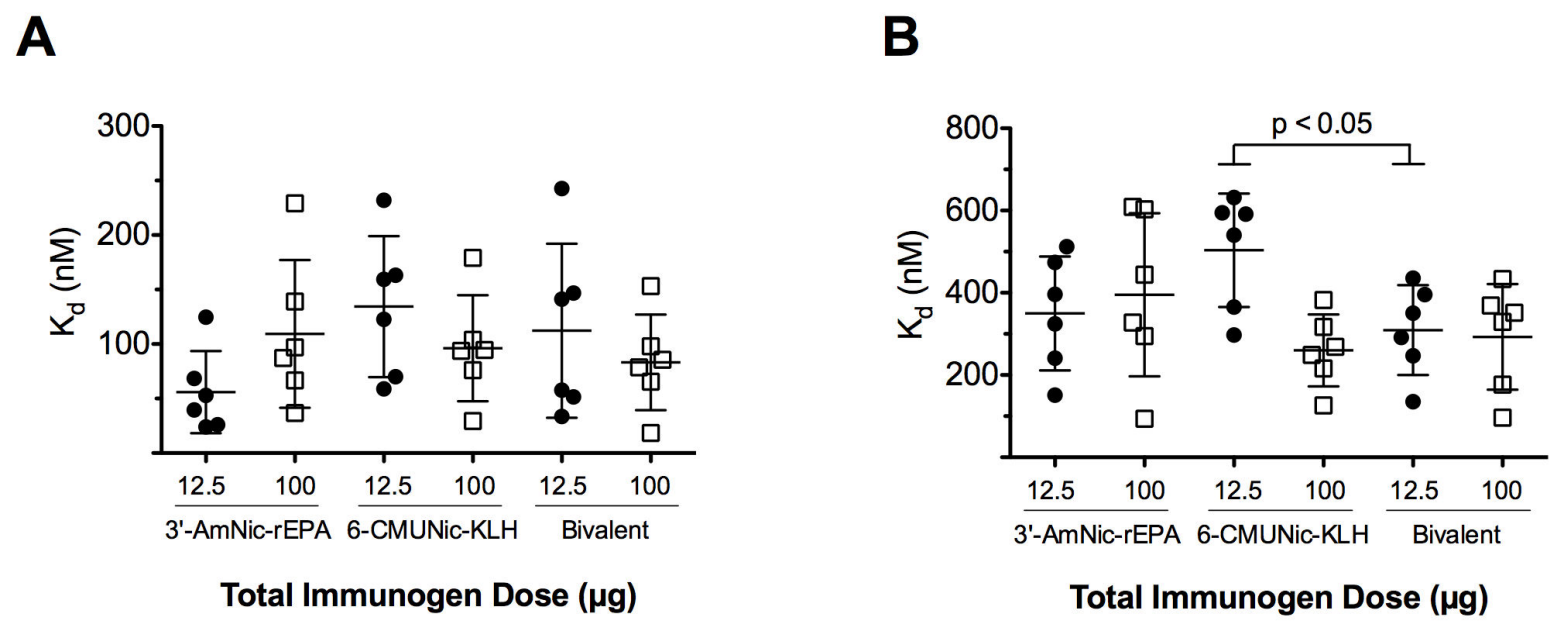
NicAb nicotine-binding affinity (K_d_) in rats immunized s.c. in alum and in rats immunized i.p. in Freund’s adjuvant (mean ± SD). NicAb affinity was preserved when immunogens were administered as components of the bivalent vaccine either s.c. in alum (A) or i.p. in Freund’s adjuvant (B). Affinity for nicotine was higher (K_d_ lower) for 6-CMUNic-KLH when administered in the bivalent vaccine i.p. in Freund’s adjuvant, but only at the 12.5 µg dose.

NicAb dissociation rate constants (K_d_) generated by vaccines administered s.c. in alum were similar regardless of immunogen dose and whether they were administered as monovalent or bivalent vaccines.

### 3.2: Experiment 2: Vaccines administered i.p. in Freund’s adjuvant

#### 3.2.1: Serum antibody titers (Figure 6A)

**Figure 6:**
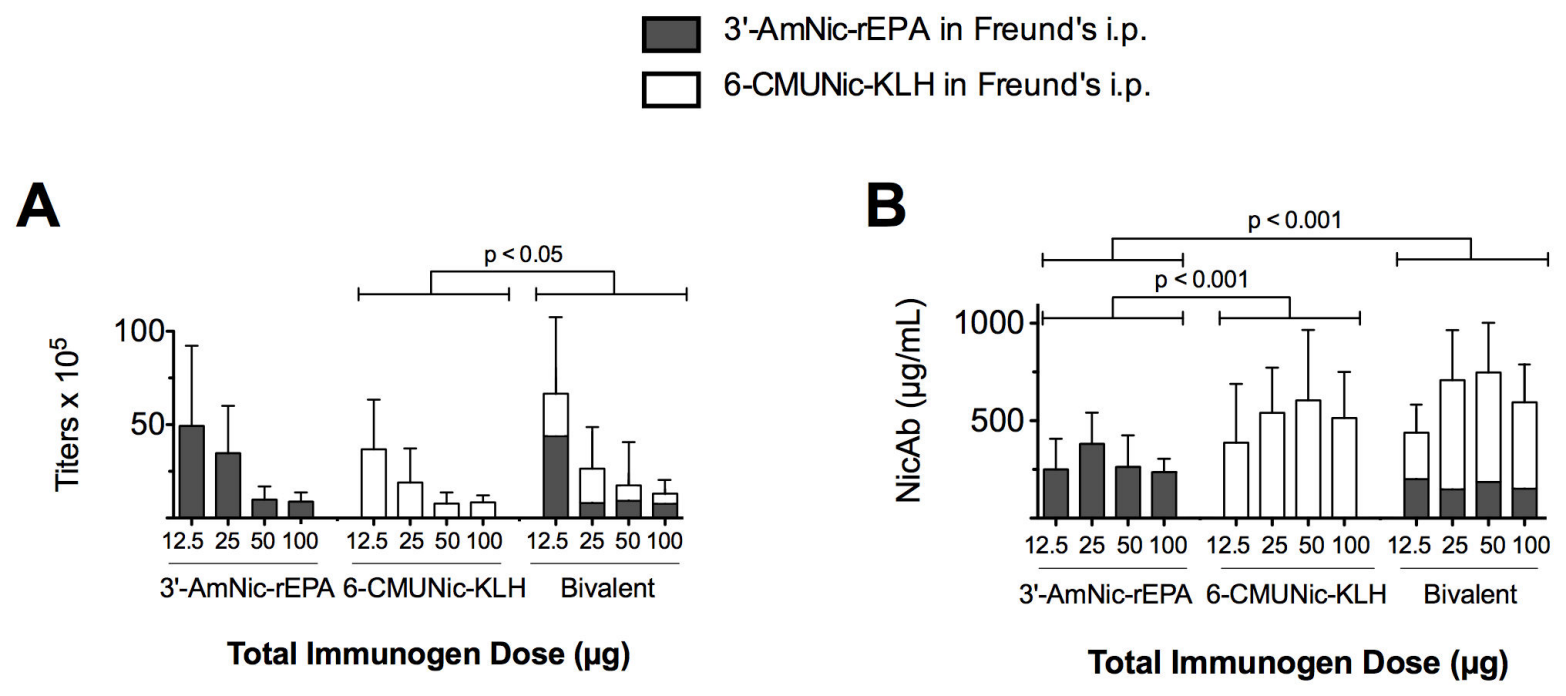
Serum nicotine-specific antibody (NicAb) titers and concentrations of rats immunized with monovalent or bivalent vaccines i.p. in Freund’s adjuvant (mean ± SD). Immunogen doses are the total administered, i.e. 100 µg bivalent = 50 µg 3′-AmNic-rEPA and 50 µg 6-CMUNic-KL H. NicAb titers (A) in the bivalent group were higher than those in the monovalent 6-CMUNic-KLH group, and NicAb concentrations (B) in the bivalent vaccine were higher than the monovalent 3′-AmNic-rEPA group.

There was a main effect of vaccine group (p < 0.05) and immunogen dose (p < 0.001) on total NicAb titer. Total NicAb titers were significantly higher in the bivalent group than in the monovalent 6-CMUNic-KLH group (p < 0.05) but not higher than in the monovalent 3′-AmNic-rEPA group. Within each vaccine group, total NicAb titers decreased with increasing total immunogen dose.

#### 3.2.2: Serum antibody concentrations (Figure 6B)

There was a main effect of vaccine group (p < 0.001) and immunogen dose (p < 0.01) on total NicAb concentration. Total NicAb concentrations in the bivalent group were significantly higher than in the monovalent 3′-AmNic-rEPA group, but were not higher than the monovalent 6-CMUNic-KLH group (p < 0.001). NicAb concentrations in the monovalent 6-CMUNic-KLH group were significantly higher than those in the monovalent 3′-AmNic-rEPA group (p < 0.001). Within vaccine groups, there were no significant effects of total immunogen dose on serum NicAb concentration.

#### 3.2.3: Contributions of individual components in bivalent group (Figure 7)

**Figure 7:**
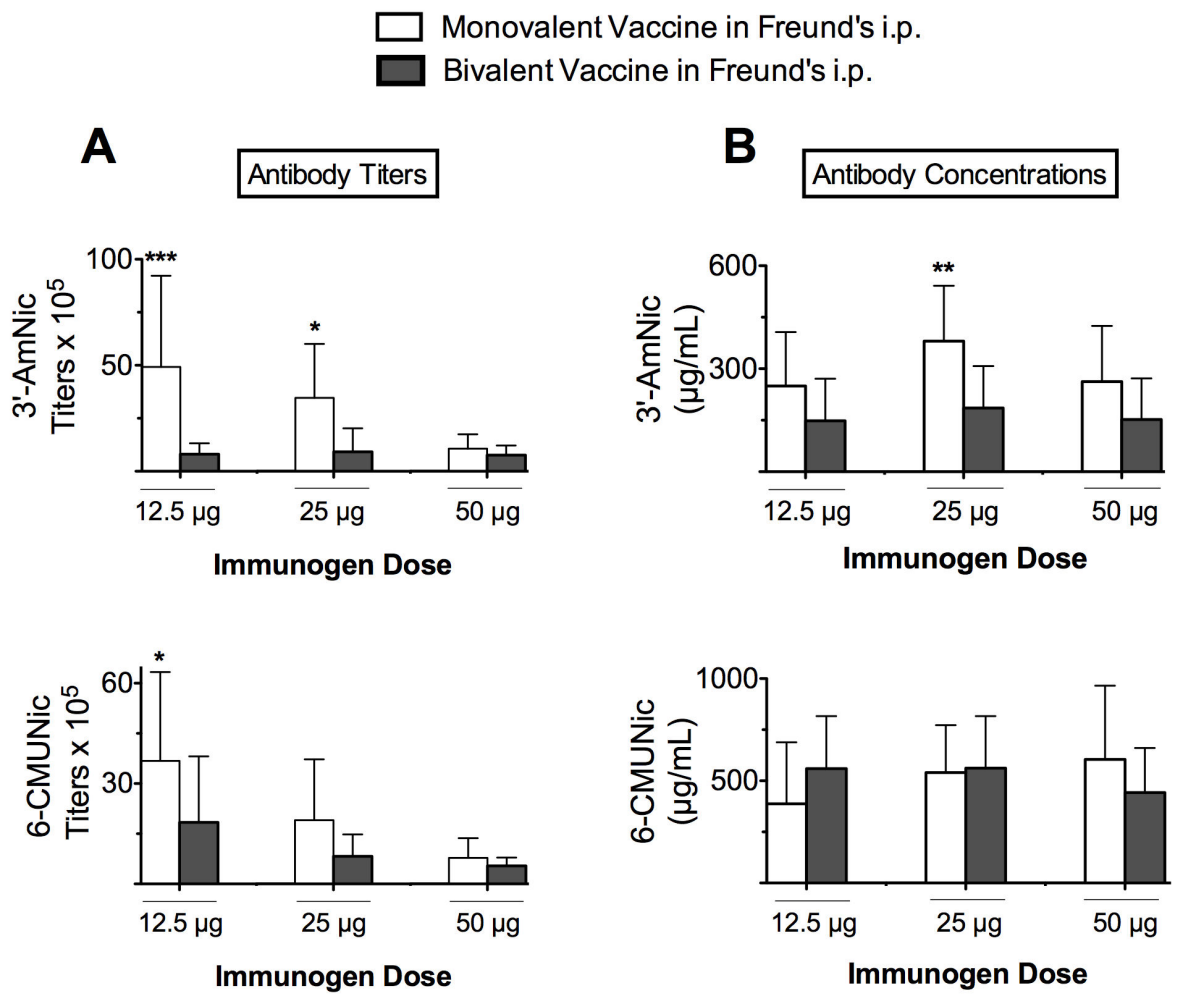
Nicotine-specific antibody titers and concentrations in monovalent vaccine groups and their individual dose-matched contributions when administered as a component of the bivalent vaccine (mean ± SD). Vaccines were administered i.p. in Freund’s adjuvant. Vertical axis indicates the hapten toward which the antibodies are directed. Note that the scales of vertical axes differ. When administered in this manner, combining immunogens impaired their immunogenicity compared to when they were administered alone. There were main effects of vaccine group on 3′-AmNic-rEPA specific titers (A) and concentrations (B), and on 6-CMUNic-KLH specific titers (A; p < 0.01 for each). Symbols show comparisons of monovalent v. bivalent vaccines at individual doses. * p < 0.05, ** p < 0.01, *** p < 0.001.

As in Figure 3, this analysis compared matched individual immunogen doses when each dose was administered alone or as a component of the bivalent vaccine (but for rats immunized i.p. with Freund’s adjuvant). In contrast to immunization s.c. in alum, some serum NicAb titers and concentrations were lower when immunogens were administered in combination. For rats receiving 3′-AmNic-rEPA, there was a main effect of vaccine group (p < 0.001) and immunogen dose (p < 0.01) on NicAb titers, and a main effect of vaccine group on NicAb concentration (p < 0.001). Rats that received 3′-AmNic-rEPA produced higher titers and concentrations when it was administered alone as a monovalent vaccine compared to when it was administered as a component of the bivalent vaccine. For rats receiving 6-CMUNic-KLH, there was a main effect of vaccine group on antibody titer (p < 0.01) but not on antibody concentration. NicAb titers were higher when 6-CMUNic-KLH was administered alone as a monovalent vaccine than when it was administered as a component of the bivalent vaccine, although this comparison was only significant at one dose. There was a main effect of 6-CMUNic-KLH dose on NicAb titer (p < 0.001).

#### 3.2.4: Antibody affinity (Figure 5B)

NicAb dissociation rate constants (K_d_) generated by vaccines administered i.p. in Freund’s adjuvant were generally similar, indicating no compromise in antibody affinity when immunogens were combined. The K_d_ of the bivalent serum represents the affinity of antibodies generated by both immunogens. 

## Discussion

Although nicotine is a small molecule, it is possible to design functionally distinct nicotine immunogens by linking carrier proteins at different positions on the nicotine molecule. The current study showed that two such nicotine immunogens can be co-administered over a range of immunogen doses without loss of activity; nicotine-specific antibody (NicAb) titers and concentrations attributable to the individual immunogens were preserved in the bivalent vaccine and mean nicotine-binding affinity of antibodies generated by the bivalent vaccine was comparable to that of the monovalent vaccines. Vaccine formulation was important, as vaccine component immunogenicity was preserved when immunogens were mixed in alum and administered s.c. but not when immunogens were mixed in Freund’s adjuvant and administered i.p. These data support the feasibility and potential benefit of combining nicotine immunogens to enhance nicotine vaccine immunogenicity.

The effect of co-administering immunogens was assessed in two ways. First, total NicAb titers and concentrations were measured to determine whether the response to the co-administered immunogens was additive. Variability in serum antibody concentrations generated by nicotine vaccines was large, making this analysis challenging. The power of this analysis was further reduced because titers and concentrations generated by 6-CMUNic-KLH were higher than those generated by 3′-AmNic-rEPA, so the contributions of the two immunogens to the total antibody response for the bivalent vaccine were not equal. 

A second planned analysis compared immunogens when administered alone as monovalent vaccines to that of the immunogens when administered in combination as a bivalent vaccine. If a single immunogen produced the same serum NicAb concentrations whether administered alone or in combination with other components, then that immunogen functioned independently. This was confirmed across all doses tested for immunogens administered s.c. in alum. Independence of immunogen responses provides the required mechanisms for an additive antibody response. 

It is reasonable to ask whether combining immunogens would be more effective than simply increasing the dose of a single immunogen, and this likely depends upon the dose-response relationship for the individual immunogen. Over the 12.5-50 μg dose range at which antibody responses generated by immunogens administered s.c. in alum individually or in combination were compared (Figure 2), there was no increase in serum antibody concentrations elicited by 3′-AmNic-rEPA alone and only a minimal increase for 6-CMUNic-KLH alone. Combining immunogens would therefore appear to present a better option for increasing the total antibody response over this dose range than simply increasing the dose either immunogen administered alone. The 100 μg (approximately 200 μg/kg) doses of monovalent immunogens did produce higher antibody levels, but this is a very high immunogen dose compared to those used in clinical trials (range approximately 1.5-6.0 μg/kg). Due to limitations in the amount of alum that can be administered clinically, it would be difficult to deliver such a high vaccine dose in humans while retaining adjuvant function. In a phase II clinical trial of 3′-AmNic-rEPA (NicVax), the highest dose tested (400 μg) was the most immunogenic but serum antibody concentrations were only 30-40% higher than those produced by 200 μg [23]. Multivalent vaccines should be most advantageous in situations such as this, where one or more immunogens produces a flat dose-response relationship.

Apart from enhancing the mean antibody response, combining vaccines might also address the challenge of poor responders (i.e. subjects achieving only minimal levels of antibody). Although limited by the number of animals per group, the current study found no significant overall correlation in the bivalent vaccine group between the concentrations of NicAbs directed against 3′-AmNic-rEPA and those directed against 6-CMUNic-KLH. Some rats with lower responses to one immunogen had better responses to the other immunogen. If the magnitude of these responses proves to be independent in larger studies, bivalent vaccines could enhance vaccine performance not just by increasing the mean antibody response but also by rescuing the overall antibody response in subjects that respond poorly to one immunogen.

Serum nicotine-specific antibody concentrations are a major determinant of their efficacy for altering nicotine distribution and effects in animals, and smoking cessation in humans [8,24,25]. Variability in serum antibody concentrations has contributed to the limited efficacy of nicotine vaccines in clinical trials. Antibody affinity for nicotine is also a major determinant of its effects on nicotine distribution in rats [21]. The current study found considerable individual variability in this measure as well, with a 4-8-fold range in the measured K_d_. This raises the possibility that individual variability in antibody affinity for nicotine may contribute to individual differences in behavioral responses to nicotine vaccines. To our knowledge, serum nicotine-binding affinities of individual animals have not been previously reported. Similarly, individual antibody affinity values in clinical trials have not been reported. Assessing variability in this measure could be beneficial to understanding vaccine response and its relationship to smoking cessation rates. 

This study examined two different vaccine formulations and routes of administration. The alum/s.c. condition was studied because of its clinical relevance; alum is the most commonly used adjuvant in human vaccines and s.c. and i.m. are the most common routes of administration. The Freund’s/i.p condition, which is not suitable for human use, was studied because it is often used in animals owing to its robust immunogenicity [26,27]. Other possibilities (alum/i.p. or Freund’s/s.c.) were not studied because our focus was to examine commonly used vaccination conditions rather than to independently assess the contributions of adjuvant and route. In contrast to observations with the alum/s.c. condition, data from immunogens administered in Freund’s/i.p. indicated interference between the immunogens at some doses when combined. Freund’s adjuvant consists of mineral oil with mycobacterium components, which is then mixed with immunogen suspended in aqueous solution to form a water-in-oil emulsion. In contrast, alum consists of an aqueous solution of aggregated Al(OH)_3_ to which immunogen is adsorbed. The adsorption of immunogen to alum may have prevented interactions between immunogen components in the bivalent vaccine formulation, whereas formulation in Freund’s did not provide this protection.

The ability of immunologically distinct vaccines to be co-administered without loss of immunogenicity is consistent with reports generated by administration of a wide range of multivalent infectious disease vaccines. Multivalent infectious disease vaccines requiring adjuvant are generally administered in alum and reports of vaccine component interference are uncommon. In a few cases interference may have been caused by incompatible preservatives or a by a non-adjuvanted component displacing an alum-adsorbed component [28,29] (for reviews, see [30,31]). These mechanisms of interference are not pertinent to the current study. We are not aware of previous reports of impaired immunogenicity for multivalent vaccines formulated in Freund’s adjuvant.

In summary, a bivalent nicotine vaccine could offer two advantages over monovalent vaccines. First, independent antibody responses to the vaccine component immunogens have the potential to generate an additive overall response. Second, individuals who respond poorly to one immunogen may respond better to the other. If so, this would reduce the number of overall poor responders with very low antibody levels. The current study focused on establishing the independence of immunogen responses under various dosing conditions and provided evidence that this approach is feasible for the immunogens studied when formulated in alum. These data support the further investigation of bi- or multivalent vaccines to improve nicotine vaccine response and efficacy.
